# Genetic and epigenetic profiling of CLL disease progression reveals limited somatic evolution and suggests a relationship to memory-cell development

**DOI:** 10.1038/bcj.2015.14

**Published:** 2015-04-10

**Authors:** E N Smith, E M Ghia, C M DeBoever, L Z Rassenti, K Jepsen, K-A Yoon, H Matsui, S Rozenzhak, H Alakus, P J Shepard, Y Dai, M Khosroheidari, M Bina, K L Gunderson, K Messer, L Muthuswamy, T J Hudson, O Harismendy, C L Barrett, C H M Jamieson, D A Carson, T J Kipps, K A Frazer

**Affiliations:** 1 Pediatrics and Rady's Children's Hospital, University of California at San Diego, La Jolla, CA, USA; 2 Moores Cancer Center, University of California at San Diego, La Jolla, CA, USA; 3 Department of Medicine, University of California at San Diego, La Jolla, CA, USA; 4 Bioinformatics and Systems Biology Program, University of California at San Diego, La Jolla, CA, USA; 5 Institute for Genomic Medicine, University of California at San Diego, La Jolla, CA, USA; 6 Department of Chemistry, Purdue University, West Lafayette, IN, USA; 7 Illumina, Inc., 5200 Illumina Way, San Diego, CA, USA; 8 Ontario Institute for Cancer Research, Toronto, Ontario, Canada; 9 Department of Medical Biophysics, University of Toronto, Toronto, Ontario, Canada; 10 Department of Molecular Genetics, University of Toronto, Toronto, Ontario, Canada; 11 Stem Cell Program, University of California San Diego, La Jolla, CA, USA

## Abstract

We examined genetic and epigenetic changes that occur during disease progression from indolent to aggressive forms of chronic lymphocytic leukemia (CLL) using serial samples from 27 patients. Analysis of DNA mutations grouped the leukemia cases into three categories: evolving (26%), expanding (26%) and static (47%). Thus, approximately three-quarters of the CLL cases had little to no genetic subclonal evolution. However, we identified significant recurrent DNA methylation changes during progression at 4752 CpGs enriched for regions near Polycomb 2 repressive complex (PRC2) targets. Progression-associated CpGs near the PRC2 targets undergo methylation changes in the same direction during disease progression as during normal development from naive to memory B cells. Our study shows that CLL progression does not typically occur via subclonal evolution, but that certain CpG sites undergo recurrent methylation changes. Our results suggest CLL progression may involve developmental processes shared in common with the generation of normal memory B cells.

## Introduction

A long-standing model of cancer evolution is that tumor cells progress through stages via a reiterative process of expansion, genetic diversification by somatic mutation, and positive selection of subclones containing specific mutations.^1^ In practice examining tumor evolution during progression from indolent to aggressive disease is challenging as multiple biopsies are required at different time points before treatment. Chronic lymphocytic leukemia (CLL) is well suited to study tumor evolution because patients are monitored closely via blood samples until symptoms necessitate treatment.^2^ In addition, CLL has been shown to often harbor multiple subclones,^3^ which could undergo selection during disease progression.^4^ A recent study used high-depth exome sequencing to examine 12 CLL patients at two time points (before and after treatment) showing clonal evolution occurring in 70% of patients,^5^ consistent with the results of other sequence-based studies.^3^ Although these studies examined fewer longitudinal CLL samples without intervening treatment, they observed that clonal evolution is less common before treatment,^3, 5^ which is consistent with the results of larger-scale CLL array profiling studies.^6^ Limitations from these studies are in the documentation of disease progression and in the limited genetic loci examined. High-depth exome sequencing studies have not yet been conducted on a large number of longitudinal samples sampled both at the time of diagnosis and immediately preceding treatment, therefore the extent and nature of clonal evolution before treatment is currently unknown.

Epigenetic modifications, including DNA methylation, have long been implicated in cancer and are suspected to be oncogenic.^7^ The polycomb repressive complex 2 (PRC2) silences genes during differentiation and is a key player in a variety of cancer types, acting by a common epigenetic modification that involves trimethylation of the amino acid lysine at position 27 in the histone protein H3 (H3K27me3).^8, 9, 10^ In cancer cells, *de novo* methylation occurs at these regions via recruitment of DNA methyltransferases by PRC2.^8^ A previous longitudinal study of CLL samples using serial samples from patients either at diagnosis and after therapy or at two time-points before treatment found that global DNA methylation may be relatively stable over time.^11^ However, an in-depth genome-wide analysis of the methylation changes that occur during CLL progression in patients from near diagnosis to when they require therapy has not yet been performed; it is unknown whether specific CpG sites recurrently undergo *de novo* methylation or whether this involves PRC2.

Here we examine 27 patients who presented with early-stage CLL disease and ultimately progressed clinically to active disease requiring treatment. We characterize somatic mutation and DNA methylation changes using paired longitudinal samples taken from these patients both at the time of diagnosis and after clinical progression, but before treatment. We show that the majority of CLL cases display-limited genetic change during that time interval, but that recurrent epigenetic changes at memory B-cell-specific PRC2 targets are associated with disease progression.

## Materials and Methods

### Sample collection

From over 900 CLL Research Consortium participants, 27 patients were chosen based on the following criteria: (1) received treatment at UCSD; (2) had a leukemia cell sample collected within approximately a year post diagnosis; and (3) had a leukemia cell sample collected within approximately a year before treatment. Twenty-six patients did not receive treatment before collection of samples at both time points, but one patient (SU77505) received one course of high-dose methylprednisolone and rituximab, to which the patient achieved a partial response and then subsequently relapsed before collection of the second sample. The treated sample was initially included in error but retained because the patient only achieved a partial response and did not appear genetically or epigenetically different from the other pretreatment leukemias. For each patient, leukemia cell samples were collected at multiple time points and frozen viably. The tumor fraction had to be at least 80% at one time-point (leukemia cells CD5+/CD19+ positive by flow cytometry) for inclusion in the study. For 19 patients germline DNA was isolated from saliva samples collected after treatment. The UCSD IRB approved the study and all subjects gave informed consent.

### sCNA predictions

Somatic copy number alterations (sCNAs) were identified in 19 of the patients by analyzing matched normal saliva and leukemia cell samples hybridized to Illumina Omni 2.5 BeadChip arrays. CNAs were predicted using the copy number prediction algorithm CNVPartition (v3.1.6; minimum probe count of 10) and verified by visual inspection. sCNAs are reported when the estimated CNV value does not equal 2 and the CNV Confidence greater than the default of 35. Additionally, sCNA regions that have been previously implicated in CLL (chr11, chr12, chr13, and chr17) were inspected visually in all samples. To obtain the number of sCNA per individual, sCNA calls near each other on the same chromosome and at multiple time points were grouped together as an sCNA group and counted as one variant. Changes between time points were assessed by visual inspection.

### Exome sequencing

Sequencing libraries were prepared and captured using SureSelect Human All Exon 50 Mb kit (Agilent Technologies, Santa Clara, CA, USA) following the manufacturer's instructions.^12^ Genomic DNA (2.5 μg) was sheared to ~175 base pairs and 100 bp paired-end reads were sequenced using the Illumina Hi Seq 2000 (San Diego, CA, USA) to 75–150 × depth. Reads were aligned to the human genome reference sequence (UCSC assembly hg19) using BWA^13^ with seed length set to 35. Duplicate reads were removed using Picard MarkDuplicate. GATK was used to realign reads around indels^14^ based on dbSNP135 and the 1000 Genomes Project indel call set. After realignment, which can create reads that have the same position as other reads, we removed the few additional duplicate reads using Picard.

### Variant calling

The single-nucleotide variants (SNVs) and indels were called using GATK v2.1-9-gb90951c UnifiedGenotyper^14^ with parameters -stand_call_conf 20.0, -stand_emit_conf 10.0 and -dcov 800. UCSC assembly hg19 and dbSNP137 were used as references. Variants were recalibrated using GATK Variant Quality Score Recalibration with metrics QD, HaplotypeScore, MQRankSum, ReadPosRankSum, FS and MQ and a 99% threshold. Somatic variants were identified using a 2 × 3 *χ*^2^-test (chisq.test in R with permutation) across germline and the two leukemia samples. Sites with a *P*-value <0.001 and a germline frequency<0.10 were called somatic.

### Identification of somatic variants with frequency changes

We performed deep-targeted sequencing using the Illumina TruSeq Custom Amplicon kit (San Diego, CA, USA) modified to include single-molecule tagging to remove duplicate reads and processed as previously described.^15^ Read coverage for each allele in each sample was identified using the Allele Count variable in GATK. Allele frequency differences were tested across leukemia samples using a 2 × 2 Fisher's Exact test and the targeted sequencing allele counts. Sites were considered to have changed if they were significantly different between samples (Benjamini–Hochberg false discovery rate (BH-FDR)<0.05).

### PyClone

PyClone (v.0.12.3) was downloaded (http://compbio.bccrc.ca/software/pyclone/) and used to estimate the number of clones in leukemia samples from each patient. Copy number estimates were imported from array data (CNVPartition calls). Allele counts were obtained from targeted resequencing data. Using validated somatic variants, samples from each patient were run together using default settings.

### Methylation arrays

We characterized changes in DNA methylation during disease progression using the Illumina HumanMethylation450 BeadChip following manufacturer instructions. Beta values were normalized and background-subtracted according to manufacturer recommended practices using GenomeStudio. Because samples were compared in pairs from the same individual, we did not remove sites that overlapped single-nucleotide polymorphisms nor process type I and type II probes separately.

### Adjustment of methylation beta values for cellular composition

Cell mixtures can be deconvoluted using differentially methylated CpGs that are cell-type specific.^16^ As the leukemia cells were not sorted we computationally corrected for methylation changes by estimating the relative composition of five cell types (B, natural killer, CD4+ T, CD8+ T and Neutrophils) and adjusted the methylation levels for the cell-type composition using linear regression. We initially observed that an unadjusted composite methylation score was positively associated with change in %CD5+/CD19+ FACS counts, suggesting an increase in sample tumor proportion during progression (Supplementary Figure 1a), which was recapitulated using just the B-cell-specific sites (Supplementary Figure 1b).^17^ To verify that the B-cell score was associated with leukemia cell load, we sorted six samples from three patients, cells were positively sorted for CD5+/CD19+ using anti-CD19 FITC microbeads multisort kit and anti-CD5 APC microbeads (MACS Miltenyi Biotec, Auburn, CA, USA) and compared the change in B-cell scores with differences in %CD5+/CD19+ cells (Supplementary Figure 1c). We further validated the B-cell score by comparing the change in mean somatic allele frequency with the B-cell methylation score (Supplementary Figure 1d). Methylation levels that were adjusted for all five cell types were used in downstream analyses.

### Changes in methylation

Adjusted and unadjusted beta values were tested for changes during progression using a Wilcoxon Signed Rank test (wilcox.test in R). For each patient, methylation values for each site in the first and second leukemia samples were paired. Sites were considered significant at BH-FDR<0.05.

### Gene set association

Genes were tested for enrichment using GO-seq,^18^ adjusting for the number of methylation probes associated with each gene.^19^ Sites were annotated to one or more genes based on the Illumina HumanMethylation450 BeadChip manifest file. Gene categories were downloaded from the Molecular Signatures Database v4.0 (http://www.broadinstitute.org/gsea/msigdb/index.jsp, msigdb.v4.0.symbols.gmt) or other references.^20, 21, 22, 23, 24^ Overlap across sites was assessed using a Fisher's Exact Test (fisher.exact in R). If previously published results were based on the 27 K array, we restricted the comparison with those CpGs in our study that were also on the 27 K array, based on Illumina Target ID.

### Histone modification enrichment analysis

ENCODE ChIP-seq peaks (405 experiments) were downloaded from UCSC (http://hgdownload.cse.ucsc.edu) on 27 February 2013. Overlaps between significantly differentially methylated probes and ENCODE ChIP-seq peaks were computed by intersecting 200 bp regions centered on significant probes with ChIP-seq peaks using bedtools intersectBed.^25^ Enrichment *P*-values were calculated using a hypergeometric test.

## Results

### CLL cells typically display-limited genetic change during disease progression

**Figure 1:**
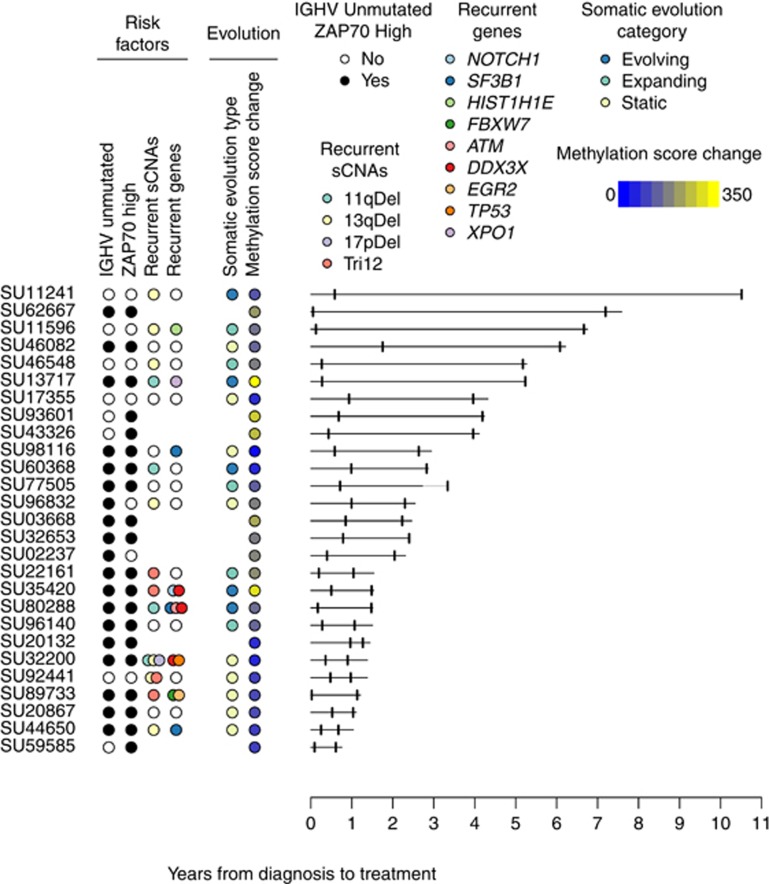
Overview of study design and results. For each patient, the years from diagnosis to treatment is shown with a solid horizontal line. The leukemia cell sample time points are shown as vertical tickmarks. For one patient (SU77505), a light grey line indicates the time from treatment to sample. The presence of established risk factors and summary of findings reported in this paper are shown in colored circles for each patient. The lack of a circle indicates missing data. Unmutated IGVH and high expression of ZAP70 are labeled in black circles. sCNAs and sSNVs that have been previously implicated in CLL are shown in colored circles according to the locus or gene. The somatic evolution type indicates whether we found that the leukemia was likely genetically clonally evolving, expanding or static during progression. The methylation score change reflects the cumulative deviation during progression at progression-associated CpGs for the specific leukemia with blue to yellow indicating a small amount of change vs a large amount.

We collected serial blood samples from 27 patients shortly after diagnosis and before they required treatment (median years between time points was 1.6 years; range 0.3–9.9 years). Our cohort included patients with both low- and high-risk prognostic factors (Figure 1). All patients presented at diagnosis with early-stage disease that progressed over time to disease requiring therapy by the International Workshop on CLL (iwCLL) criteria.^2^

**Figure 2:**
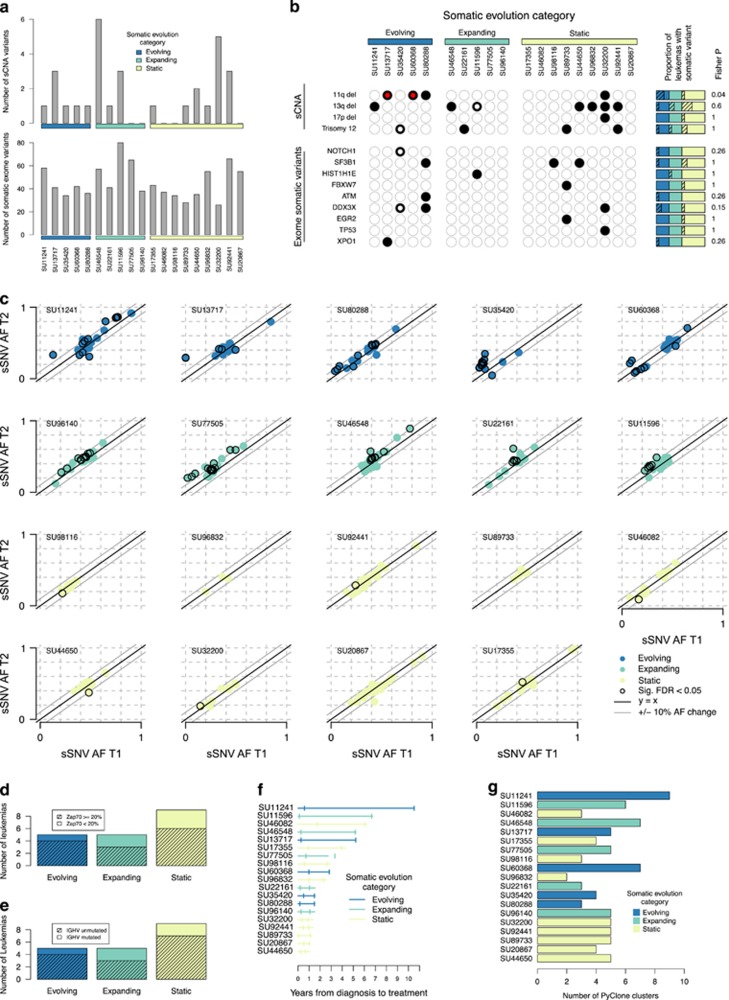
Somatic evolution in CLL. Only patients with germline DNA (*N*=19) are shown. (**a**) Barplot of the number of sCNAs and the number of sSNVs identified in either of the leukemia cell samples from a patient, grouped by somatic evolution type. (**b**) Recurrently mutated sSNVs and sCNAs. A black dot indicates that either of the leukemia cell samples carried the sCNA or sSNV. A red circle in the back dot indicates that the variant arose in the second sample and was not apparent in the first. A white circle inside the black dot indicates that the variant was present in both samples, but appeared to change during progression by manual inspection for sCNAs or significantly by Fisher's Exact test at FDR<0.05 for SNVs. The hatched areas in the rectangles on the right summarize the relative proportion of leukemia samples in each evolution type with the given somatic mutation, with the Fisher *P*-value indicating if the somatic mutation is more prevalent in evolving vs the other two categories. (**c**) Somatic allele frequencies in first leukemia cell sample (*x* axis) and second leukemia cell sample (*y* axis), as identified from deep-targeted sequencing. Points are circled in black if they were significantly different between the two samples (FDR<0.05). Plots are color coded according to pattern of somatic evolution. The central black line indicates *y*=*x*, with the flanking grey lines indicating a change of 10%. (**d** and **e**) The hatched areas show the relative proportion of leukemia samples that are Zap70 positive and IGHV unmutated, respectively. (**f**) Timeline of disease progression. Horizontal line indicates time from diagnosis to treatment. Tickmarks indicate point where sample was taken. (**g**) Number of genetic clusters, as estimated by PyClone.

We identified somatic copy number alterations (sCNAs), detecting 30 sCNAs in 19 patients corresponding to an average of 1.7 sCNAs genome-wide per patient (range, 0–6; Figure 2a and Supplementary Table 1). sCNAs previously implicated in CLL were common with 13/19 (68%) of patients showing an sCNA in at least 1 of 4 recurrently altered loci (Figure 2b and Supplementary Table 1). The majority of sCNAs were observed at both sample collection time points. However, we observed two instances of 11q deletions and one instance of a novel sCNA (SU13717) that were only present in the latter sample (Figure 2b and Supplementary Table 1). These results are in agreement with studies showing that over 80% of CLL cases have sCNA that are frequently subclonal.^26^

We identified somatic point mutations through whole-exome sequencing of matched germline and leukemia cell samples at both clinical time points. In total, we identified 871 somatic SNVs (sSNVs) corresponding to 26–80 coding or splice site point mutations per patient (Figure 2a, Supplementary Table 2). We identified indels in three genes (to increase confidence indel analysis was restricted to known recurrently mutated genes in CLL; Supplementary Table 2). We validated the somatic mutation calls by deep-targeted sequencing (~850 × coverage) using the Illumina TruSeq Custom Amplicon (TSCA) kit (San Diego, CA, USA) modified to account for PCR duplicates.^15^ We interrogated 521/871 (60%) of the sSNVs and validated 484/521 (93%) of these sites (Supplementary Table 3). The vast majority of validated sSNVs were observed at both time-points with only one leukemia case displaying mutations unique to the second time-point—a deletion in *NOTCH1* and an sSNV in *DDX3X*. Although 13/871 of the sSNVs we observed were in recurrently mutated genes (Figure 2b, Supplementary Table 2), the vast majority were novel and likely passenger mutations.^3^

To examine subclonal patterns of change during tumor progression, we analyzed the 484 validated sSNVs (mean allelic fraction of 39% (range 0–97%) at the first time point) for significant changes in allele frequency during disease progression. Overall, 109/484 (23%) of the sites changed significantly (FDR<0.05), of which only 37/109 (34%) had a >10% allele frequency change (Figure 2c and Supplementary Table 3). Because the CLL cells were not sorted, some changes in allele frequency during progression could be due to the leukemia expanding relative to the normal mononuclear cells. To differentiate this from clonal evolution where one subclone is increasing in frequency in comparison to other subclone(s), we defined three different categories: (1) evolving, where at least one sSNV increased and at least one decreased in frequency (*N*=5); (2) expanding, where two or more sSNVs increased in frequency and none decreased (*N*=5); and (3) static, where no or only one sSNV increased or decreased in frequency (*N*=9; Figure 2c). Evolving cases had an average median allele frequency change of ~9% (range 6–14%) and on average ~44% (range 17–88%) sSNVs changed frequency. Expanding cases had an average median allele frequency change of ~7% (range 5–9%) and on average ~28% (range 18–42%) sSNVs that changed frequency (Supplementary Table 4). Thus the absolute changes in sSNV frequencies across the two-sample collection time points were comparable between the evolving and expanding leukemia cell populations, but in the former particular subclones were expanding and others contracting, whereas in the latter the leukemia was likely expanding relative to the normal population. These data show that in ~25% of CLL cases, subclonal somatic evolution does occur with new sCNAs and point mutations occurring or significantly changing in frequency, but in most cases, mutations are present at the time of diagnosis and show limited change in their relative frequencies.

### CLL somatic evolution category and prognostic risk factors

Although we are limited by sample size, we explored if significant relationships exist between the somatic evolution categories and prognostic factors, time to progression, presence of driver mutations or extent of intraclonal genetic heterogeneity. Patients with CLL cells that use unmutated immunoglobulin heavy chain (IGH) variable region (IGHV) and/or express high ZAP-70 generally have a more aggressive clinical course;^27^ however, such prognostic factors were not associated with a particular category of CLL (Figures 2d and e). Time to treatment was also not associated (Figure 2f). We also do not observe a strong association with the presence of somatic mutations in nine genes previously shown to be recurrent in CLL and/or high-risk sCNAs with any one of the three different categories (Supplementary Table 2, Figure 2b). However, we observed a nominal association between the 11q deletion and the evolving category compared to the combined group of expanding and static leukemias (*P*=0.04, unadjusted *P*-value). Only two mutations in driver genes significantly changed allele frequencies, both in the same individual (SU35420) (Figure 2b). We additionally examined the relationship between CLL category and intraclonal heterogeneity estimated using PyClone.^28^ We observed a total of 90 subclones with each leukemia case carrying an average of 5 (range 2–9) subclones (Figure 2g). However, we observed no statistically significant difference between evolving leukemias vs. expanding and static leukemias combined (Mann–Whitney *P*=0.4). These results suggest that some somatic variants, such as 11q deletions, may be associated with somatic evolution category, but larger sample sizes will be required to define associations that are statistically significant.

### CLL cases show methylation changes at specific CpG sites during disease progression

**Figure 3:**
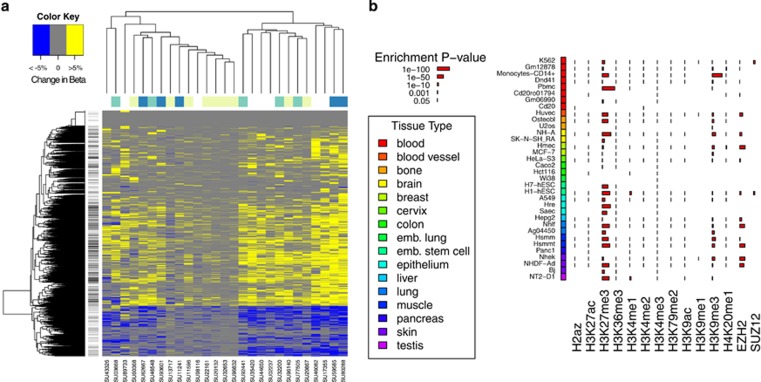
Methylation differences associated with progression implicate PRC2. (**a**) Heatmap of methylation changes at significant loci. Cells are colored according to whether the methylation of the represented CpG site increased by at least 5% (yellow), changed between -5 to 5% (grey), or decreased by at least 5% (blue). If germline DNA was available (*N*=19), the somatic evolution type is shown along the top (yellow=static, teal=expanding, blue=evolving). Sites that are within 100 bp of an H3K27me3-marked region in the most associated ENCODE PBMC sample (see Figure 3b) are shown as black lines next to the row dendrogram. (**b**) Sites that showed significant differences were tested for whether they were likely to be near regions bound by modified histones or bound by the EZH2 and SUZ12 proteins as measured by ChiP-seq in a variety of cell types using the ENCODE data. Significant enrichment is indicated with a red bar. If no mark is present, the assay was not performed.

We profiled the leukemia cell samples from 27 patients at both clinical time points using Illumina HumanMethylation450 BeadChip arrays (450k arrays). After adjusting methylation levels for cellular composition (see Methods), we identified 4,752 CpG sites co-located near 2,670 genes that showed significant changes in the leukemia cells during progression (Supplementary Table 5). We did not observe clustering of methylation patterns at these 4,752 CpGs sites associated with time to progression or somatic evolution category. Instead, they appeared heterogeneous with each individual having a different combination of sites affected (Figure 3a). Considering all patients, the majority of sites (3670/4752, 77%) showed modest increases in methylation (median increase of 2.9%, range 0.5–9.5%). Changes for a given patient were also modest, with 49 sites on average changing more than 20% and 1220 changing more than 5% (Figure 3a). The leukemias with the least amount of methylation changes were of the static evolution category and from patients that had progressed relatively quickly after diagnosis (Figures 1 and 3a). However, there were instances of static disease with longer times between diagnosis and treatment that had high numbers of changes, indicating that methylation changes do not require genetic subclonal evolution. Although leukemias with the highest numbers of CpG sites that changed were in the expanding or evolving categories, there were leukemias in each of the three categories that had relatively low numbers of methylation changes. These results indicate that methylation changes occurring at the 4752 progression-associated CpGs are modest in effect size, heterogeneous across individuals and are not specific to a certain somatic evolution category.

### CpGs associated with CLL progression are near PRC2 target genes in B cells

To assess the biological relevance of the methylation changes at the 4752 progression-associated CpGs, we performed gene set analysis using a method that corrects for the biased representation of probes to genes on the 450k arrays.^19^ We observed significant differences between CpG sites that increase or decrease in methylation (Figure 3a) during CLL progression; those that increase are strongly enriched near genes associated with H3K27me3 modifications and PRC2 targets (Supplementary Table 6), whereas those that decrease show no significant relationship. Further analysis showed that the CpGs most commonly experiencing methylation changes were significantly clustered around genes with H3K27me3-marked promoters in naive B-cells (*P*=1 × 10^−33^), germinal center B-cells (*P*=9 × 10^−21^) and embryonic stem cells (*P*=3 × 10^−41^; Supplementary Table 6). They also were clustered around genes bound by EZH2 in germinal center B-cells (*P*=3 × 10^−29^) and hESCs (*P*=1 × 10^−23^).^20, 29^ These results show that the PRC2 is bound normally in B-cells near CpGs that undergo hypermethylation during disease progression in patients with CLL. We further explored colocalization between the 4572 progression-associated CpGs and chromatin modifications within ±100 bp of a ChIP-seq peak using 406 experiments by the ENCODE project.^30^ We observed strong enrichment of regions marked by H3K27me3 and H3K9me3, and bound by PRC2 components SUZ12 and EZH2 (Figure 3b).

We compared our findings with those of previous methylation studies of CLL and aging. The promoters of several genes known to be methylated in CLL samples (*SFRP1*, *SFRP2*, *DAPK1*, *CDH1*) had CpG sites that were significantly associated with disease progression (Supplementary Table 5).^31, 32^ We did not, however, observe changes in methylation in *ZAP70*, indicating that this prognostic marker does not apparently change over time.^33^ We examined the overlap with genes associated with CpG sites that show changes in the CLL mouse model of *TCL1A* overexpression and observed a significant overlap with 23/51 genes that showed changes in this mouse model near a progression-associated site (*P*=0.0002) identified in our study.^21^ Because methylation changes at Polycomb associated sites has been linked to cellular aging, we examined the overlap between CLL progression-associated sites and age-associated sites from three studies.^22, 23, 24^ We observed little overlap with 353 CpGs that show age-acceleration in cancer tissues (4/353 significant, Fisher *P*=0.56) or 96 CpGs found to be associated with age in blood (2/96 markers, Fisher *P*=0.24).^22, 24^ We did, however, observe an overlap between progression-associated sites and age-associated sites that are enriched for Polycomb target promoters (8/207 markers, Fisher *P*=0.001).^23^ These data demonstrate that the CpGs associated with CLL progression frequently overlap genes previously shown to be differentially methylated in CLL, and that changes in methylation status are unlikely to be solely due to increased cellular age or cellular composition changes associated with aging.

### Progression-associated CpGs show methylation changes similar to those in normal memory B-cell development

**Figure 4:**
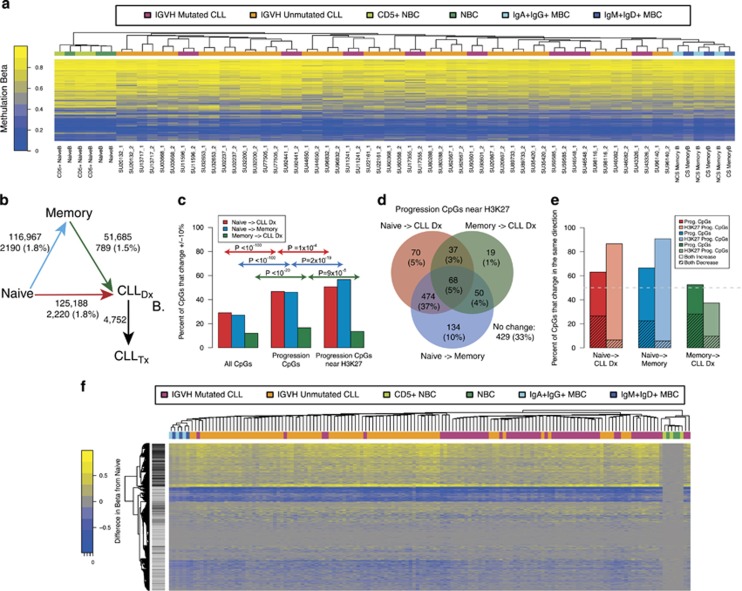
Methylation changes at progression-associated CpGs show similarities to memory B cells. (**a**) Methylation levels for CLL samples at progression-associated CpGs, grouped with naive B cells (CD5+ and normal) and memory B cells (IgA+IgG+ and IgM+IgD+). IGHV mutation status of CLL samples is shown along the top according to the legend colors. (**b**) Schematic representation showing the number of CpG sites that differ by at least 10% between the average of the six memory B-cells, the six naive B cells, and the 27 CLL samples taken at the first time point. The number of CpGs that also change during progression are reported under the number and as a percent of the sites that differ. (**c**) Barplot showing the enrichment of progression-associated CpGs near H3K27me3 marks and all progression-associated CpGs compared with all CpGs that differ at least 10% between comparisons of the three cell types. *P*-values were calculated using the hypergeometric test based on the counts in each category. (**d**) Venn diagram showing the overlap of progression-associated sites that were near H3K27me3 regions and also differed at least 10% between comparisons of cell types shown in 4B. (**e**). Barplots showing the percent of CpGs that change methylation status in the same direction (both increase or both decrease) during progression (CLL_DX_ to CLL_TX_) as between comparisons of the cell-types in 4B. (**f**). Heatmap showing methylation Beta levels from the ICGC data at progression-associated CpGs. Methylation levels are shown as the difference between the sample and the average of six naive B-cell samples. Black and grey lines indicate which rows are near H3K27me3 regions (black) or not (grey).

We compared the methylation levels at the 4752 progression CpGs of the 27 paired leukemia samples in our study to that of six naive (CD5+ and normal) and six memory B cells (class switched and nonclass switched) using 450 k array data from the International Cancer Genome Consortium (ICGC).^34^ Using hierarchical clustering, we observed that in all patients the methylation status at these CpG loci were more similar to that of memory B cells than to that of naive B cells (Figure 4a). We then examined whether methylation changes at the 4752 progression-associated CpGs were similar to changes that happen during leukemia initiation and/or normal B-cell development. To do this, we identified CpGs that differed by at least ±10% between naive, memory and the first CLL time point and identified those that overlapped with progression CpGs (Figure 4b). Considerably more CpGs change methylation status between naive and memory B-cells or between naive B cells and the first CLL samples then between memory B cells and the first CLL time point. However, we observed a significant enrichment for progression CpGs in total (and even more so for those near H3K27me3) with sites that differed between pair-wise comparisons of all three cell types (naive B cells, memory B cells and the first CLL sample) as compared with all CpGs (Figure 4b). For progression CpGs near H3K27me3, we observed a strong overlap between the sites that differed between naive and memory and those that differed between naïve and the first CLL sample (Figure 4d). For these overlapping sites, methylation changes during progression and differences between naive and memory and naive and CLL first time point were largely in the same direction (Figure 4e), particularly for CpGs near H3K27me3 that become more methylated during progression. These data show that the majority of CpGs that undergo methylation change during CLL disease progression undergo changes in the same direction during normal memory B-cell development.

Previous work suggested that CLL with mutated or unmutated IGHV are derived from memory and naive B cells, respectively. However, we did not observe an overlap between 3265 CpGs associated with IGHV mutation status and the 4752 progression-associated sites (*P*=0.64).^34^ These findings suggest that although the CpGs that undergo methylation changes during progression overlap those that change during the development from naive to memory cells, or transition from naive to CLL, they are not the same as those that differ between IGHV-mutated and unmutated CLL or those that are change during the transition from memory cells to CLL.

As the leukemia cell samples from the 27 patients in our study were not sorted we adjusted methylation levels for cellular composition computationally; therefore, we performed additional analyses using sorted cells to validate our findings. To examine changes during progression, we positively sorted serial samples from three patients for CD5+/CD19+ cells and performed 450 k arrays. We averaged the change in methylation during progression and identified sites that changed the most (top and bottom 5% quantiles). We compared these with the progression-associated sites and we observed positive enrichment (OR=1.35, *P*=1.1 × 10^−11^), showing that progression-associated CpGs are likely to show changes in sorted CLL cells during progression. In addition, sites in the top 5% quantile were enriched for sites near H3K27me3 regions (OR=1.79, *P*=3.7 × 10^−238^). To test whether sorted CLL cells look more similar to memory B cells at progression-associated sites, we examined the methylation levels of the 139 sorted CLL cells in the ICGC dataset relative to naive and memory B cells (Figure 4f). We observed clustering within CLL according to IGVH mutation status; however, both types of CLL were more similar to memory B cells than naive at these 4752 sites. In addition, progression-associated sites near H3K27me3 regions predominantly showed higher methylation in the CLL and memory B cells compared with naive. These results provide an important control and suggest that our findings based on data from compositionally corrected CLL samples are valid in sorted CLL cells.

## Discussion

This study provides insight into the genetic and epigenetic changes that occur during progression from an indolent form of cancer to a physiologically more aggressive form. Our findings are inconsistent with the dogma of cancer progression through stages via somatic mutation followed by positive selection of subclones containing specific mutations.^1^ In contrast to evolution documented in pre- vs post-treated samples,^3^ we observed little clonal evolution during progression from indolent to aggressive disease, suggesting that changes observed in treated samples may arise during repopulation of the cancer and are not required for the leukemia to become more aggressive.

Many of the tumors that showed static somatic evolution and low methylation score changes progressed to require treatment quickly (<2 years). It is possible that these tumors were aggressive at the point of diagnosis and showed no or undetectable further genetic and epigenetic changes during progression. Because these patients progressed clinically between the sample time points, however, this suggests that clinical progression can occur with little genetic or epigenetic change.

We observed coincident changes in DNA methylation associated with Polycomb repression across the majority of CLL cases. PRC2 target methylation in cancer is common;^8^ however, the methylation patterns observed are usually similar to those in stem cells.^35^ The changes that we observe occur more often at sites that undergo epigenetic modifications during normal differentiation of naive to memory B cells. This suggests that clinical progression from indolent to active forms of CLL may involve developmental processes shared in common with those involved in normal B-cell differentiation.

In the 26% of leukemia cases that do show clonal evolution, there may be somatic drivers involved, such as *NOTCH1* or chr11 deletions (including the interval encoding *ATM*), as has been suggested by previous work.^36^ We observed three instances of *11q* deletions occurring in leukemias that showed clonal evolution, with two instances arising during progression, findings consistent with a late-acting role for *ATM.* Both *ATM* and *TP53* are DNA damage response genes recurrently mutated or deleted in CLL and associated with poor prognosis. We hypothesize that effects of *ATM* and *TP53* mutations may be through the PRC2. ATM-mediated phosphorylation of EZH2 has been shown to reduce protein stability, reducing PRC3 formation and increasing H3K27me3 in cells with ATM deficiency.^37^ TP53, which is downstream of ATM, has also been linked to H3K27me3 through one of its targets, the lincRNA *LINC-PINT*.^38^ These new roles for ATM and TP53 that are unrelated to their DNA damage response functions may help to explain how mutations in these genes are advantageous for CLL cells despite the very low rates of somatic mutation in CLL and little evidence of clonal selection pre-treatment.

In summary, we have traced the molecular changes at genetic and epigenetic levels in CLL cases as they clinically progress from indolent to active disease requiring therapy. We show that CLL progression does not typically occur via genetic clonal evolution, but that that certain CpG sites undergo methylation consistent with an increase in PRC2 activity. Our results suggest that changes in epigenetic regulation via the PRC2 occur during CLL progression and may involve developmental processes shared in common with the generation of normal memory B cells. Interestingly, memory B cells share hematopoietic stem cell features that may be advantageous to CLL, including the ability to undergo long-term self-renewal.^39^ Further research into the role of PRC2 and how it interacts with recurrently mutated genes in CLL may provide insight into the molecular mechanisms underlying CLL disease progression.

## Notes

KLG is an employee of Illumina and may own stock in the company. The remaining authors declare no conflict of interest.

## References

[bib1] Greaves M Maley CC Clonal evolution in cancer Nature 2012 481 306 313 22258609 10.1038/nature10762 PMC3367003

[bib2] Hallek M Cheson BD Catovsky D Caligaris-Cappio F Dighiero G Dohner H Guidelines for the diagnosis and treatment of chronic lymphocytic leukemia: a report from the International Workshop on Chronic Lymphocytic Leukemia updating the National Cancer Institute-Working Group 1996 guidelines Blood 2008 111 5446 5456 18216293 10.1182/blood-2007-06-093906 PMC2972576

[bib3] Landau DA Carter SL Stojanov P McKenna A Stevenson K Lawrence MS Evolution and impact of subclonal mutations in chronic lymphocytic leukemia Cell 2013 152 714 726 23415222 10.1016/j.cell.2013.01.019 PMC3575604

[bib4] Burrell RA McGranahan N Bartek J Swanton C The causes and consequences of genetic heterogeneity in cancer evolution Nature 2013 501 338 345 24048066 10.1038/nature12625

[bib5] Ojha J Ayres J Secreto C Tschumper R Rabe K Van Dyke D Deep sequencing identifies genetic heterogeneity and recurrent convergent evolution in chronic lymphocytic leukemia Blood 2015 125 492 498 25377784 10.1182/blood-2014-06-580563 PMC4296010

[bib6] Braggio E Kay NE VanWier S Tschumper RC Smoley S Eckel-Passow JE Longitudinal genome-wide analysis of patients with chronic lymphocytic leukemia reveals complex evolution of clonal architecture at disease progression and at the time of relapse Leukemia 2012 26 1698 1701 22261920 10.1038/leu.2012.14 PMC3893815

[bib7] Timp W Feinberg AP Cancer as a dysregulated epigenome allowing cellular growth advantage at the expense of the host Nat Rev Cancer 2013 13 497 510 23760024 10.1038/nrc3486 PMC4636434

[bib8] Schlesinger Y Straussman R Keshet I Farkash S Hecht M Zimmerman J Polycomb-mediated methylation on Lys27 of histone H3 pre-marks genes for de novo methylation in cancer Nat Genet 2007 39 232 236 17200670 10.1038/ng1950

[bib9] Mack SC Witt H Piro RM Gu L Zuyderduyn S Stutz AM Epigenomic alterations define lethal CIMP-positive ependymomas of infancy Nature 2014 506 445 450 24553142 10.1038/nature13108 PMC4174313

[bib10] Ben-Porath I Thomson MW Carey VJ Ge R Bell GW Regev A An embryonic stem cell-like gene expression signature in poorly differentiated aggressive human tumors Nat Genet 2008 40 499 507 18443585 10.1038/ng.127 PMC2912221

[bib11] Cahill N Bergh AC Kanduri M Goransson-Kultima H Mansouri L Isaksson A 450K-array analysis of chronic lymphocytic leukemia cells reveals global DNA methylation to be relatively stable over time and similar in resting and proliferative compartments Leukemia 2013 27 150 158 22922567 10.1038/leu.2012.245

[bib12] Tewhey R Nakano M Wang X Pabon-Pena C Novak B Giuffre A Enrichment of sequencing targets from the human genome by solution hybridization Genome Biol 2009 10 R116 19835619 10.1186/gb-2009-10-10-r116 PMC2784331

[bib13] Li H Durbin R Fast and accurate long-read alignment with Burrows-Wheeler transform Bioinformatics 2010 26 589 595 20080505 10.1093/bioinformatics/btp698 PMC2828108

[bib14] DePristo MA Banks E Poplin R Garimella KV Maguire JR Hartl C A framework for variation discovery and genotyping using next-generation DNA sequencing data Nat Genet 2011 43 491 498 21478889 10.1038/ng.806 PMC3083463

[bib15] Smith EN Jepsen K Khosroheidari M Rassenti LZ M DA Ghia EM Biased estimates of clonal evolution and subclonal heterogeneity can arise from PCR duplicates in deep sequencing experiments Genome Biol 2014 15 420 25103687 10.1186/s13059-014-0420-4 PMC4165357

[bib16] Houseman EA Accomando WP Koestler DC Christensen BC Marsit CJ Nelson HH DNA methylation arrays as surrogate measures of cell mixture distribution BMC Bioinformatics 2012 13 86 22568884 10.1186/1471-2105-13-86 PMC3532182

[bib17] Calvanese V Fernandez AF Urdinguio RG Suarez-Alvarez B Mangas C Perez-Garcia V A promoter DNA demethylation landscape of human hematopoietic differentiation Nucleic Acids Res 2012 40 116 131 21911366 10.1093/nar/gkr685 PMC3245917

[bib18] Young MD Wakefield MJ Smyth GK Oshlack A Gene ontology analysis for RNA-seq: accounting for selection bias Genome Biol 2010 11 R14 20132535 10.1186/gb-2010-11-2-r14 PMC2872874

[bib19] Geeleher P Hartnett L Egan LJ Golden A Raja Ali RA Seoighe C Gene-set analysis is severely biased when applied to genome-wide methylation data Bioinformatics 2013 29 1851 1857 23732277 10.1093/bioinformatics/btt311

[bib20] Velichutina I Shaknovich R Geng H Johnson NA Gascoyne RD Melnick AM EZH2-mediated epigenetic silencing in germinal center B cells contributes to proliferation and lymphomagenesis Blood 2010 116 5247 5255 20736451 10.1182/blood-2010-04-280149 PMC3012542

[bib21] Chen SS Raval A Johnson AJ Hertlein E Liu TH Jin VX Epigenetic changes during disease progression in a murine model of human chronic lymphocytic leukemia Proc Natl Acad Sci USA 2009 106 13433 13438 19666576 10.1073/pnas.0906455106 PMC2726368

[bib22] Horvath S DNA methylation age of human tissues and cell types Genome Biol 2013 14 R115 24138928 10.1186/gb-2013-14-10-r115 PMC4015143

[bib23] Teschendorff AE Menon U Gentry-Maharaj A Ramus SJ Weisenberger DJ Shen H Age-dependent DNA methylation of genes that are suppressed in stem cells is a hallmark of cancer Genome Res 2010 20 440 446 20219944 10.1101/gr.103606.109 PMC2847747

[bib24] Weidner CI Lin Q Koch CM Eisele L Beier F Ziegler P Aging of blood can be tracked by DNA methylation changes at just three CpG sites Genome Biol 2014 15 R24 24490752 10.1186/gb-2014-15-2-r24 PMC4053864

[bib25] Quinlan AR Hall IM BEDTools: a flexible suite of utilities for comparing genomic features Bioinformatics 2010 26 841 842 20110278 10.1093/bioinformatics/btq033 PMC2832824

[bib26] Dohner H Stilgenbauer S Benner A Leupolt E Krober A Bullinger L Genomic aberrations and survival in chronic lymphocytic leukemia N Engl J Med 2000 343 1910 1916 11136261 10.1056/NEJM200012283432602

[bib27] Rassenti LZ Huynh L Toy TL Chen L Keating MJ Gribben JG ZAP-70 compared with immunoglobulin heavy-chain gene mutation status as a predictor of disease progression in chronic lymphocytic leukemia N Engl J Med 2004 351 893 901 15329427 10.1056/NEJMoa040857

[bib28] Roth A Khattra J Yap D Wan A Laks E Biele J PyClone: statistical inference of clonal population structure in cancer Nat Methods 2014 11 396 398 24633410 10.1038/nmeth.2883 PMC4864026

[bib29] Ku M Koche RP Rheinbay E Mendenhall EM Endoh M Mikkelsen TS Genomewide analysis of PRC1 and PRC2 occupancy identifies two classes of bivalent domains PLoS Genet 2008 4 e1000242 18974828 10.1371/journal.pgen.1000242 PMC2567431

[bib30] Raney BJ Cline MS Rosenbloom KR Dreszer TR Learned K Barber GP ENCODE whole-genome data in the UCSC genome browser (2011 update) Nucleic Acids Res 2011 39 D871 D875 21037257 10.1093/nar/gkq1017 PMC3013645

[bib31] Raval A Tanner SM Byrd JC Angerman EB Perko JD Chen SS Downregulation of death-associated protein kinase 1 (DAPK1) in chronic lymphocytic leukemia Cell 2007 129 879 890 17540169 10.1016/j.cell.2007.03.043 PMC4647864

[bib32] Seeliger B Wilop S Osieka R Galm O Jost E CpG island methylation patterns in chronic lymphocytic leukemia Leuk Lymphoma 2009 50 419 426 19347729 10.1080/10428190902756594

[bib33] Claus R Lucas DM Ruppert AS Williams KE Weng D Patterson K Validation of ZAP-70 methylation and its relative significance in predicting outcome in chronic lymphocytic leukemia Blood 2014 124 42 48 24868078 10.1182/blood-2014-02-555722 PMC4125353

[bib34] Kulis M Heath S Bibikova M Queiros AC Navarro A Clot G Epigenomic analysis detects widespread gene-body DNA hypomethylation in chronic lymphocytic leukemia Nat Genet 2012 44 1236 1242 23064414 10.1038/ng.2443

[bib35] Widschwendter M Fiegl H Egle D Mueller-Holzner E Spizzo G Marth C Epigenetic stem cell signature in cancer Nat Genet 2007 39 157 158 17200673 10.1038/ng1941

[bib36] Fegan C Robinson H Thompson P Whittaker JA White D Karyotypic evolution in CLL: identification of a new sub-group of patients with deletions of 11q and advanced or progressive disease Leukemia 1995 9 2003 2008 8609709

[bib37] Li J Hart RP Mallimo EM Swerdel MR Kusnecov AW Herrup K EZH2-mediated H3K27 trimethylation mediates neurodegeneration in ataxia-telangiectasia Nat Neurosci 2013 16 1745 1753 24162653 10.1038/nn.3564 PMC3965909

[bib38] Marin-Bejar O Marchese FP Athie A Sanchez Y Gonzalez J Segura V Pint lincRNA connects the p53 pathway with epigenetic silencing by the Polycomb repressive complex 2 Genome Biol 2013 14 R104 24070194 10.1186/gb-2013-14-9-r104 PMC4053822

[bib39] Luckey CJ Bhattacharya D Goldrath AW Weissman IL Benoist C Mathis D Memory T and memory B cells share a transcriptional program of self-renewal with long-term hematopoietic stem cells Proc Natl Acad Sci USA 2006 103 3304 3309 16492737 10.1073/pnas.0511137103 PMC1413911

