# Supplementary material for: Genetic and epigenetic profiling of CLL disease progression reveals limited somatic evolution and suggests a relationship to memory-cell development
Source: Blood Cancer J. 2015 Apr 10;5(4):e303–. doi: 10.1038/bcj.2015.14 (PMC4450323; doi:10.1038/bcj.2015.14)
Supplement: Supplementary Information [file bcj201514x1.pdf]

Supplementary Table Titles

Supplementary Table 1. List of sCNA segments

Supplementary Table 2. List of sSNV and somatic indel calls from exome sequencing data

Supplementary Table 3. List of sSNV calls from targeted sequencing data

Supplementary Table 4. Summary of targeted sequencing

Supplementary Table 5. Significantly changing CpG sites using methylation data adjusted for B-cell composition

Supplementary Table 6. Gene set enrichment for genes annotated to progression-associated CpG sites

Supplementary Figure

Supplementary Figure 1

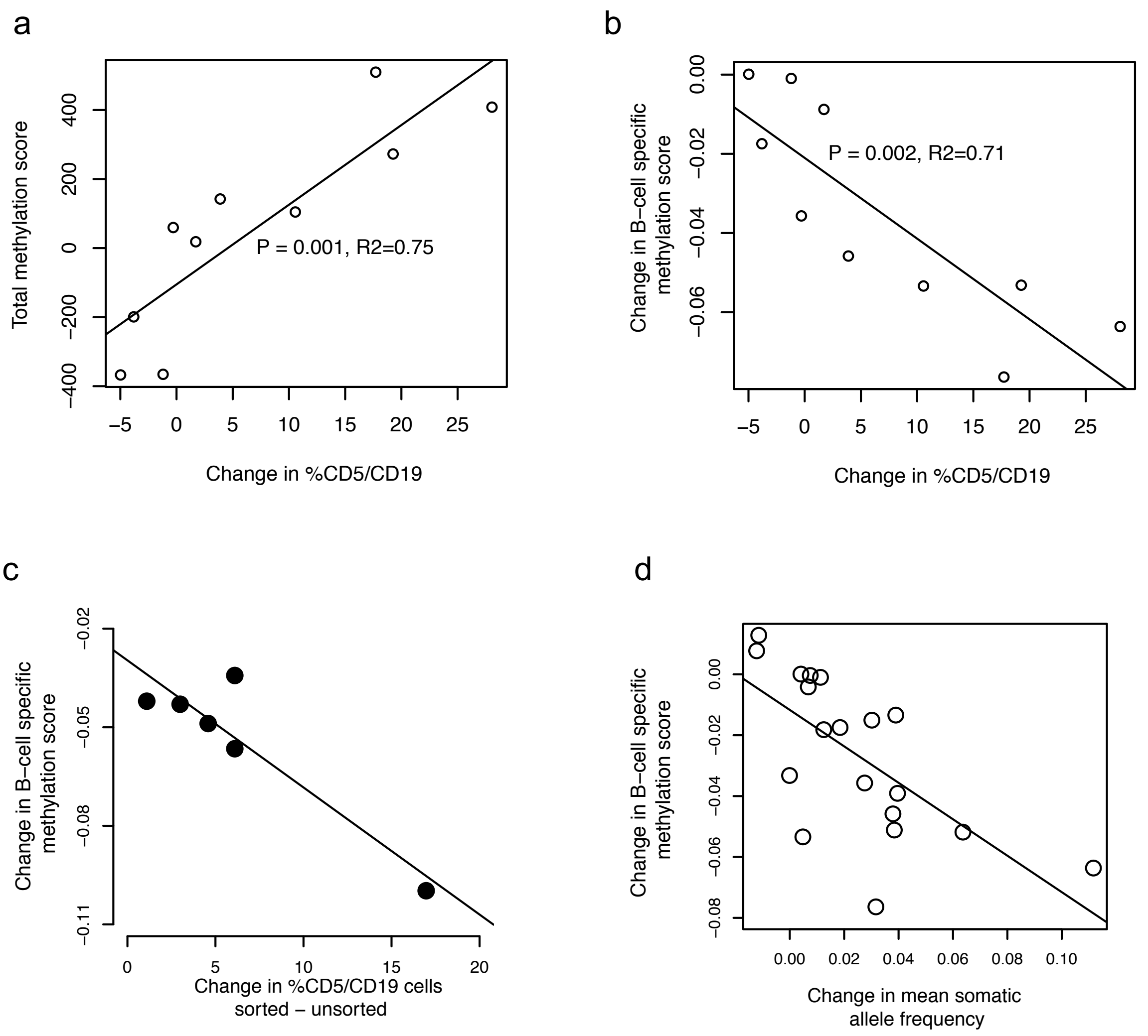

Figure Legend for Supplementary Figure

Supplementary Figure 1. Increases in leukemia cell proportions drive methylation differences. A) To capture the overall pattern of methylation change prior to adjusting for cell composition, we developed a total methylation score consisting of the sum across all progression-associated sites (from unadjusted analyses) of the difference between the

sample's β and the mean β, multiplied by -1 if the site decreased on average. We plot the change in this total score against the change in %CD5+/CD19+ FACS counts between the two clinical time-points in ten of the CLL cases and show a significant positive relationship by linear regression with P-value and R2 shown. B) A cell-type specific score was then calculated for each sample defined as the average methylation level of sites that have been shown to be cell type specifically de-methylated in each of 5 cell types (B, Natural Killer, CD4+ T, CD8+ T, and Neutrophils). We show the relationship between the change in the B-cell score to changes in %CD5+CD19+ FACS counts. As the B-cell score decreases, more B-cells would be expected to be present, consistent with an increase in the %CD5+CD19+ counts. C) For 6 samples from 3 patients, we magnetically labeled and sorted CLL cells by positive selection of CD19+/CD5+ cells using anti-CD19 FITC microbeads multisort kit and anti-CD5 APC microbeads (MACS Miltenyi Biotec, Auburn, CA). We plot the change in B-cell scores before and after sorting and show that they are correlated with differences in %CD5+/CD19+ cells, consistent with the change in B-cell score representing changes in leukemia-cell load. D) We plot the change in the B-cell methylation score in all samples that also underwent exome seqeuencing and show that a decrease in B-cell methylation score correlates with an increase in mean somatic allele frequency, consistent with expanding leukemia increasing in frequency relative to the normal population.
